# Enhancement of Cognitive Function by Andrographolide-Loaded Lactose β-Cyclodextrin Nanoparticles: Synthesis, Optimization, and Behavioural Assessment

**DOI:** 10.3390/ph17070966

**Published:** 2024-07-21

**Authors:** Debashish Paramanick, Kagithala Naga Rani, Vijay Kumar Singh, Parakh Basist, Rahmuddin Khan, Jameel H. Al-Tamimi, Omar M. Noman, Mansour N. Ibrahim, Abdulsalam Alhalmi

**Affiliations:** 1 School of Medical and Allied Science, Galgotias University, Greater Noida 203201, India; debashishparamanick02@gmail.com; 2 School of Pharmacy, Rawatpura Sarkar University, Raipur 492015, India; vijaysingh1207@gmail.com; 3 School of Medical and Allied Sciences, K.R. Mangalam University, Gurugram 122103, India; parakhbasistbnpl85@gmail.com; 4 Department of Pharmaceutics, School of Pharmaceutical Education and Research, Jamia Hamdard, New Delhi 110062, India; rkm.hamdard@gmail.com (R.K.); asalamahmed5@gmail.com (A.A.); 5 Department of Zoology, College of Science, King Saud University, P.O. Box 2455, Riyadh 11451, Saudi Arabia; 6 Department of Pharmacognosy, College of Pharmacy, King Saud University, P.O. Box 2457, Riyadh 11451, Saudi Arabia; onoman@ksu.edu.sa; 7 Department of Agricultural Engineering, College of Food and Agriculture Sciences, King Saud University, Riyadh 11451, Saudi Arabia; malsamee@ksu.edu.sa

**Keywords:** memory loss, andrographolides, behavioural models, nanoparticles, cyclodextrin, animals

## Abstract

This study investigates whether Andrographolide-loaded Lactose β-Cyclodextrin (ALN-βCD) nanoparticles enhance cognitive function, particularly spatial learning and memory. The successful conjugation of lactose to β-cyclodextrin was confirmed via 1H NMR spectroscopy, facilitating neuronal cell entry. The solvent evaporation method was used to create the nanoparticles, which were characterised for particle size, PDI, zeta potential, and drug release. The nanoparticles exhibited a size of 247.9 ± 3.2 nm, a PDI of 0.5 ± 0.02, and a zeta potential of 26.8 ± 2.5 mV. FTIR and TEM analyses, along with in vitro drug release and BBB permeability studies, confirmed their stability and efficacy. Behavioural tests, including the Elevated Plus Maze, Y-Maze, Object Recognition, and Locomotor Activity tests, demonstrated significant improvements in memory, motor coordination, and exploration time in the nanoparticle-treated groups. The group treated with ALN-βCD at a dose of 100 mg/kg/p.o. showed superior cognitive performance compared to the group receiving free andrographolides (AG). Biochemical assays indicated a significant reduction in acetylcholinesterase activity and lipid peroxidation, suggesting increased acetylcholine levels and reduced oxidative stress. Histopathological examination showed improved neuronal function without toxicity. The results showed significant improvements (*p* < 0.001) in memory and cognitive abilities in experimental animals, highlighting the potential of ALN-βCD nanoparticles as a non-invasive treatment for memory loss. These promising findings warrant further exploration through clinical trials.

## 1. Introduction

Few effective treatments exist for neurological diseases and disorders around the world. This includes conditions such as Parkinson’s, schizophrenia, migraines, and Alzheimer’s disease (AD). AD, a severe neurological disease, causes memory loss and cognitive deterioration [1]. The great majority of AD cases are sporadic and most often found in persons 65 and older [2]. Aggregation and deposition of Amyloid-Beta (Aβ) plaques are likely the main cause of AD. New research demonstrates that AD is caused by more than oxidative stress and neuroinflammation [3,4]. Current basic pharmacological treatments cannot cure or halt AD. The Food and Drug Administration recently approved anticholinesterase medicines donepezil, tacrine, galantamine, and rivastigmine for symptom therapy [5]. These therapies may cause headaches, vertigo, nausea, vomiting, and lack of appetite.

**Figure 1:**
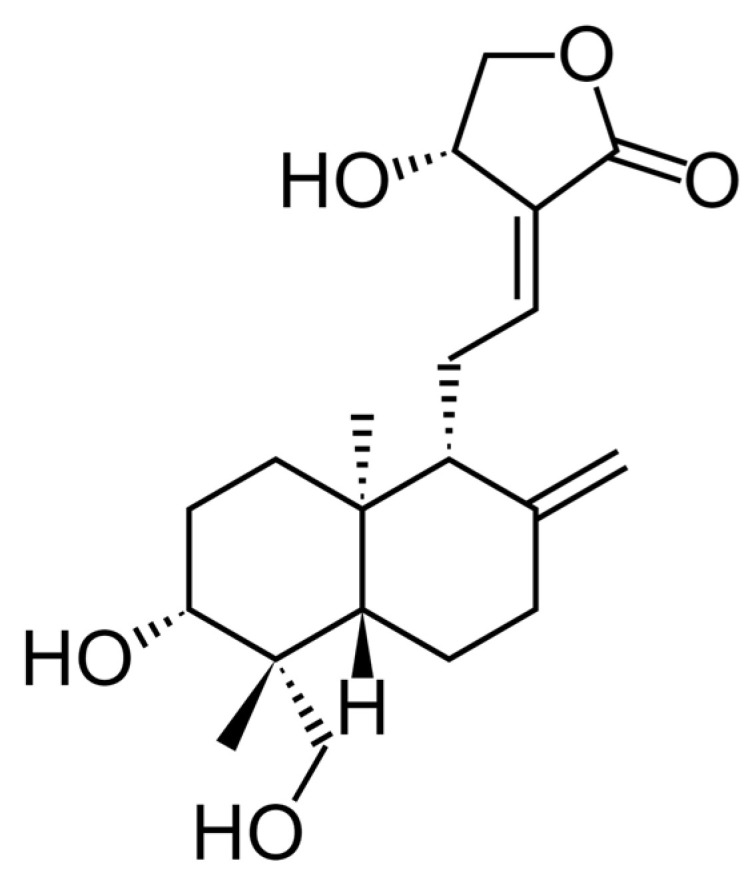
Structure of andrographolides.

Phytotherapeutics are attractive due to their diverse medical efficacies and decreased toxicity as compared to synthetic drugs. Andrographis paniculata Nees is a popular Asian Acanthaceae medicine [6]. Andrographolide (AG), neoandrographolide, 14-deoxyandrographolide, and 14-deoxy-11-12-didehydroandrographolide are the main active components of Herba Andrographidis, which are bicyclic diterpenes with a γ-lactone moiety (Figure 1) [7]. By blocking nuclear factor kappa B (NF-Kb) and mitogen-activated protein kinase (MAPK) signalling, which cause inflammation and apoptosis. Traditionally used for therapy, Andrographis paniculata has many pharmacological properties. It prevents inflammation-mediated neurodegeneration, dopamine-mediated neurotoxicity, nicotine-induced brain oxidative stress, and cerebral ischemia [8]. A recent study by Serrano and colleagues found that AG prevented Aβ oligomer damage in lab tests, reduced Aβ and tau phosphorylation in mice, controlled amyloid plaque development, and restored spatial memory in AβPP/PS1 mice, a transgenic model for AD [9].

Due to its weak water solubility and volatility, AG has minimal bioavailability and limited clinical use [10]. Drug delivery techniques including oral micro-emulsions, cyclodextrin inclusion complexes, and liposomes improve AG bioavailability. Due to their low loading capacity, low encapsulation efficacy, and poor stability, these approaches limit AG distribution and accumulation in the body after delivery [11]. 

There is currently no evidence that AG’s blood–brain barrier (BBB) permeability might potentially lead to therapeutically significant quantities of the chemical in the central nervous system [12]. Since the physiochemical and biomimetic characteristics of the carrier determine BBB penetration, rather than the qualities of the pharmaceuticals put into nanovectors and concealed, nano-sized delivery systems may offer adequate drug permeability into the brain [13].

The study aimed to enhance cognition in animals by improving BBB permeability with Andrographolide-loaded Lactose β-Cyclodextrin (ALN-βCD) nanoparticles through receptor-mediated transport. Behavioural models were selected to provide insights into spatial learning, memory, anxiety, and fear-related behaviours, making them powerful tools in neuroscience and behaviour studies. Motor coordination and locomotor activity provided insights for muscle strength and stimulant activity.

## 2. Results and Discussion

### 2.1. Synthesis of Lactose-Appended β-Cyclodextrin

**Figure 2:**
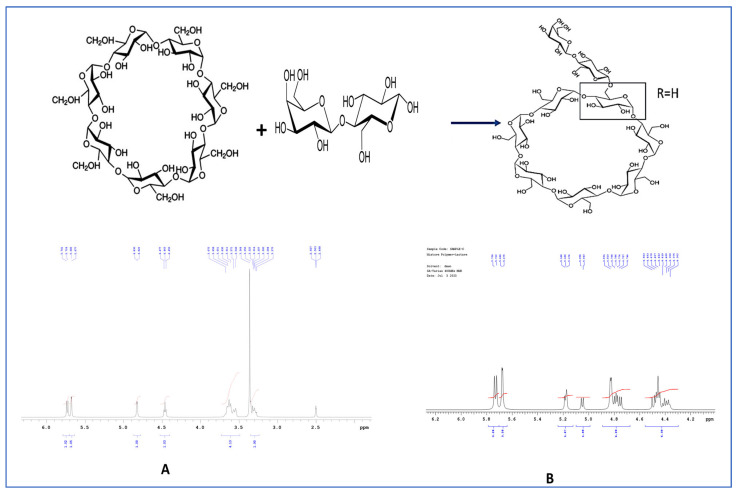
NMR spectrum of (**A**) β-cyclodextrin and (**B**) lactose-appended β-cyclodextrin.

The proton nuclear magnetic resonance (1H NMR) spectrum of lactose-appended β-cyclodextrin showed a chemical shift of the anomeric proton of lactose, which appeared at *δ* 5.675– 5.739, indicating a 27% yield (Supplementary Figure S1). Figure 2 shows the number of peaks for the anomeric proton of β-cyclodextrin, indicating that one lactose molecule is appended to each β-cyclodextrin molecule. Because β-cyclodextrin (CD) is unable to enter neuronal cells after crossing the blood–brain barrier, lactose has been included to improve the solubility and bioavailability of the CD complex. Lactose can enter neuronal cells with the assistance of the glucose transporter, which is present in various locations within the brain, such as neurons, astrocytes, and the capillaries of endothelial cells. However, the lactose-CD complex is not intended to be transferred through the glucose transporter due to its large size [14]. 

### 2.2. Andrographolides Loaded Lactose β-Cyclodextrin Nanoparticles (ALN-βCD Nanoparticles)

The prepared formulation particle size was 247.9 ± 3.2 nm, which is an important parameter for brain drug delivery because a small particle size allows a higher amount of drug to be delivered into the brain while also increasing the rate of drug absorption due to the larger surface area. The PDI value of the nanoparticles was nearly 0.5 ± 0.02, indicating that the nanoparticles have a narrow size distribution. Another parameter, the zeta potential of ALN-βCD nanoparticles, was found to be −26.8 ± 2.5 mV, which indicates a high level of stability, effective dispersion, and high repulsion between particles, preventing aggregation of nanoparticles and allowing them to remain suspended for a longer time. The TEM of the prepared nanoparticles shows a spherical shape (Supplementary Figure S2). The bulk of drug delivery system nanoparticles are 100–300 nm in size, as numerous researchers have claimed that this is a crucial characteristic for BBB permeability [15]. However, these findings indicated that the ALN-βCD nanoparticles employed in this study effectively maintain their stability and prevent aggregation, retaining their nanoscale size. Overall, the ALN-βCD nanoparticles demonstrated favourable properties, including small particle size, narrow size distribution, high stability, and resistance to aggregation. These characteristics make them promising candidates for brain-targeted drug delivery systems, potentially enhancing the therapeutic efficacy of andrographolides.

### 2.3. Optimization of Formulation

**Figure 3:**
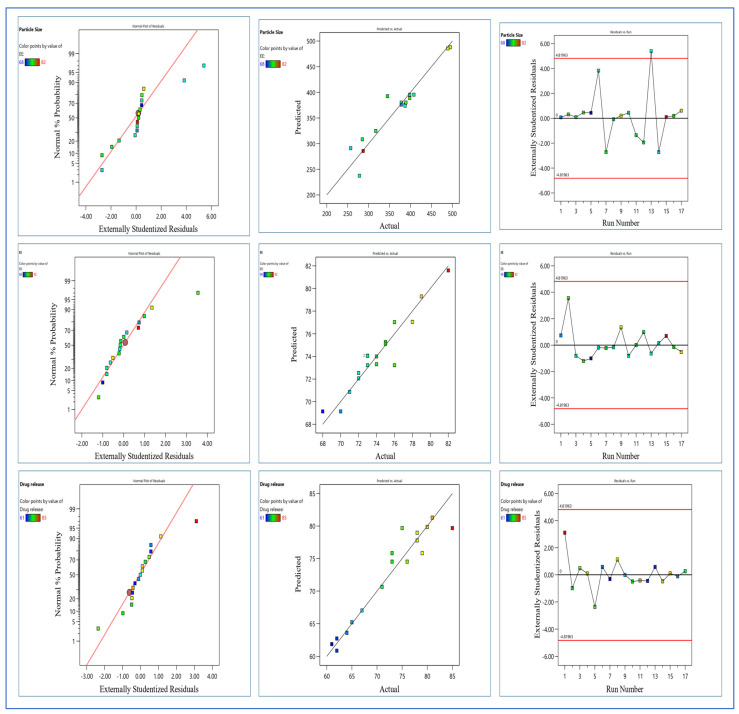
Diagnostic plots of nanoparticles for particle size, entrapment efficacy (EE), and % drug release.

A design expert was used as a novel tool for achieving optimization and minimizing variability while obtaining high-quality products with uniform particle size and stability. The Box-Behnken design was used to look at how the independent variables, such as particle size, entrapment efficiency, and percentage drug release, interact with each other and with quadratic effects. Three factors with three levels of low (−1), high (+1), and intermediate (0) have been used. The independent variables of polymer concentration (A), surfactant concentration (B), and sonication time (C) generated 17 experimental runs by the Box-Behnken design (shown in Table 1), and diagnostic plots of the formulation (shown in Figure 3) represent properties such as normal % probability, externally studentised residuals vs. predicted plot of particle size, entrapment efficiency %, and % drug release.

**Figure 4:**
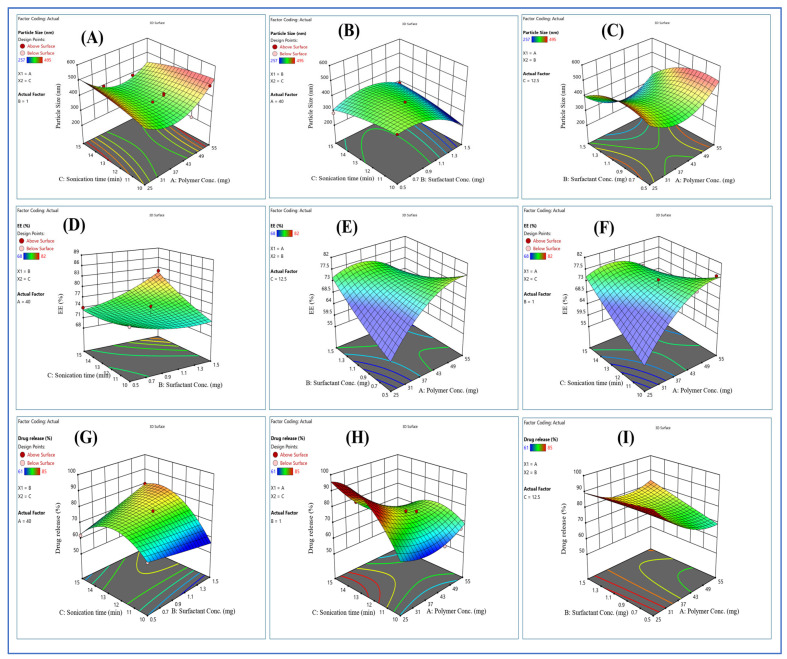
Three-D response surface plots. (**A**–**I**) Comparative effects of surfactant conc., polymer conc., and sonication time on particle size (**A**–**C**), EE (**D**–**F**), and % drug release (**G**–**I**).

Figure 4 shows three-dimensional response surface morphology, which shows the effects of independent variables (polymer concentration, surfactant concentration, and sonication time) on dependable variables (particle size, entrapment efficacy, and percent drug release) [16,17].

The high and low levels of independent variables were determined based on initial laboratory observations. A trial-and-error approach to selecting the formulation’s excipients ensured the formulation’s durability and consistency. The best-fitting model was quadratic with the highest coefficient of correlation (i.e., R^2^ = 1). The particle size of nanoparticles ranged from 257 to 495 nm, and the coefficient of correlation in a quadratic model of particle size gave an adjusted R^2^ value of 0.8014. The drug release from the nanoparticles ranged from 61 to 85% via a quadratic model, and the adjusted R^2^ value was 0.8093. The EE between 70 and 82% via quadratic model adjusted R^2^ value was 0.8146. The mathematical quadratic equations drawn from the experimental design for the calculation of particle size, EE, and % drug release are shown below.

Particle Size = +380.46 + 0.5275 × A − 52.84 × B − 1.55 × C − 25.20 A × B − 40.37 A × C + 41.48 B × C 111.35 × A^2^ − 66.32 × B^2^ − 15.52 × C^2^

EE = +73.57 + 3.00 × A + 1.36 × B + 1.88 × C − 7.15 A × B − 7.54 A × C + 2.78 B × C − 6.00 × A^2^ + 1.27 × B^2^ + 0.7359 × C^2^

Drug release = +77.34 − 8.10 × A + 2.07 × B + 5.25 × C + 4.96 A × B − 9.62 A × C + 6.49 B × C + 6.76 × A^2^ + 0.1715 × B^2^ − 11.46 × C^2^

### 2.4. FTIR Spectroscopy 

FTIR spectroscopy characterised the chemical stability of the encapsulated nanoparticles in the core of the polymer. The structure of AG shows a C4 primary group (CH_2_–OH) and furan ring, which assume importance because of their bioactivity. Stretching mode calculation 1741.42 cm^−1^ has a potential energy distribution. The polymer demonstrates a characteristic peak at 3339 cm^−1^ due to the O–H group stretching. An intense peak at 2851 cm^−1^ due to asymmetric/symmetric stretching was also seen in the free AG drug sample. The peak at 2851 cm^−1^ disappeared (Supplementary Figure S3) in the nanoparticle, indicating that the drug was encapsulated in the polymeric core.

### 2.5. Drug Loading and Entrapment Efficacy

Drug loading and entrapment efficacy were measured in Table 2 at different concentrations (5 µg to 30 µg) of AG in a nanoparticle. The resulting AG concentration is directly proportional to drug entrapment, whereas drug loading decreases. The drug entrapment percentage remained relatively constant between 74 ± 0.45% and 82 ± 032%, thus indicating a significant amount of the drug was successfully encapsulated in the nanoparticle. The drug loading percentage decreased from 26% to 17% as the drug concentration increased, indicating that a lower amount of the drug was required to achieve the same drug entrapment.

### 2.6. In Vitro Drug Release

**Figure 5:**
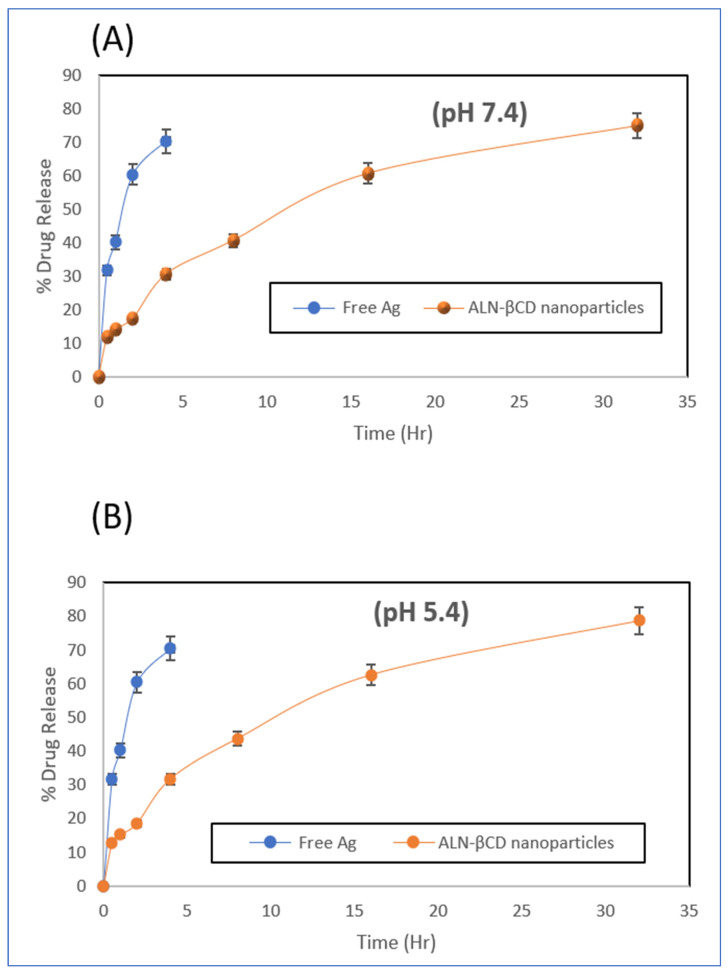
In vitro drug release at pH 7.4 (**A**) and pH 5.4 (**B**).

The in vitro drug release of nanoparticles was analysed by measuring the concentration of the drug at various time intervals. The results shown in Figure 5 indicate that the concentration of the drug increased gradually over time, with an initial cumulative % release. The drug from the nanoparticle was released in a similar pattern at both pH levels, i.e., 75.08% and 78.73% at pH 7.4 and 5.4, respectively, at 32 h.

### 2.7. Cytotoxicity Assay 

**Figure 6:**
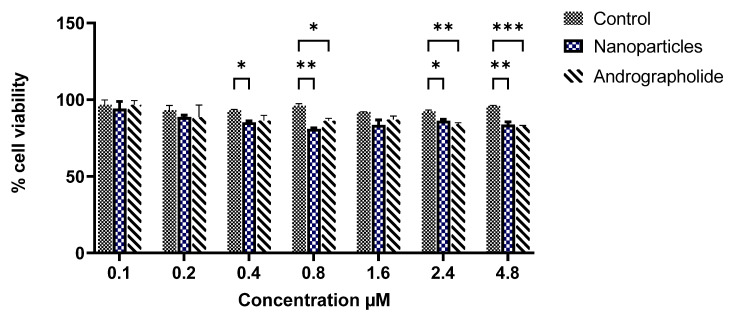
Percentage cell viability of prepared nanoparticles. *** *p* < 0.001. ** *p* < 0.1. * *p* < 0.05.

The cell viability of free AG and ALN-βCD nanoparticles on hCMEC/D3 cell lines at different concentrations (0.1 µM, 0.2 µM, 0.4 µM, 0.8 µM, 1.6 µM, 2.4 µM, 4.8 µM) is shown in Figure 6. The results indicated that cells incubated with AG and ALN-βCD nanoparticles at a low concentration (0.1 µM) showed almost the same viability as the control. The cell viability slowly decreased with increasing concentration. However, the viability was still 80% at the high concentration of 4.8 µM. Thus, cytotoxicity is dose dependent in manner [18]. 

### 2.8. In Vitro BBB Permeability

**Figure 7:**
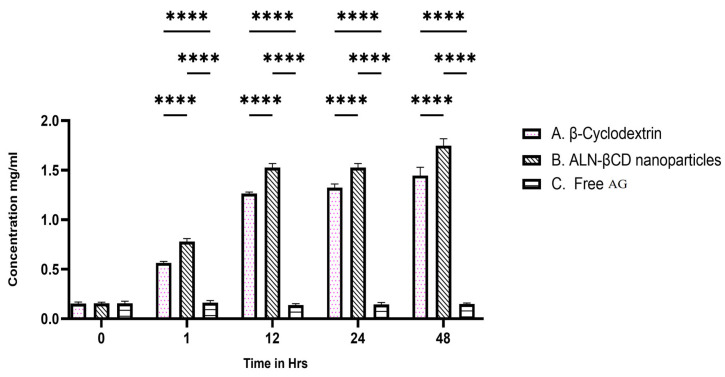
In vitro permeability of prepared nanoparticles across the BBB. **** *p* < 0.001.

The in vitro BBB permeability of three different formulations, namely free AG, ALN-βCD nanoparticles, and β-cyclodextrin, was assessed based on fluorescence units measured at an excitation/emission wavelength of 490/525 nm. The results obtained for each formulation are presented in Figure 7. The results indicate that the nanoparticles exhibited the highest fluorescence units, suggesting higher permeability across the BBB in vitro. Free AG showed lower fluorescence units, indicating comparatively lower permeability. β-Cyclodextrin demonstrated an intermediate level of fluorescence units, whereas ALN-βCD nanoparticles showed a high level of fluorescence, suggesting good permeability across the BBB. These in vitro results provide an initial indication of the formulations’ BBB permeability.

### 2.9. In Vitro Cellular Uptake

**Figure 8:**
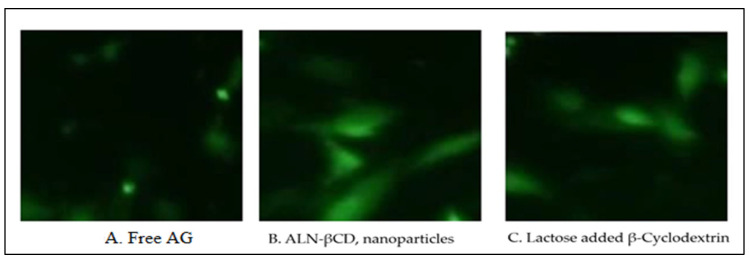
Cellular uptake of prepared nanoparticles. Free AG (**A**), ALN- βCD nanoparticles (**B**) and Lactose added β-Cyclodextrin (**C**).

The cellular uptake of ALN-βCD nanoparticles, free AG, and lactose-appended β-cyclodextrin was measured and compared. The data obtained for cellular uptake are presented in the results. Figure 8 illustrates that cellular uptake was significantly higher for ALN-βCD nanoparticles compared to free AG and lactose-appended β-cyclodextrin. These findings suggest that ALN-βCD nanoparticles have a greater potential for cellular uptake and can potentially be utilised as effective carriers for drug delivery applications.

### 2.10. Assessment of Behavioural Activity

#### 2.10.1. Assessing the Elevated Plus Maze’s (EPM) Capacity to Improve Memory

**Figure 9:**
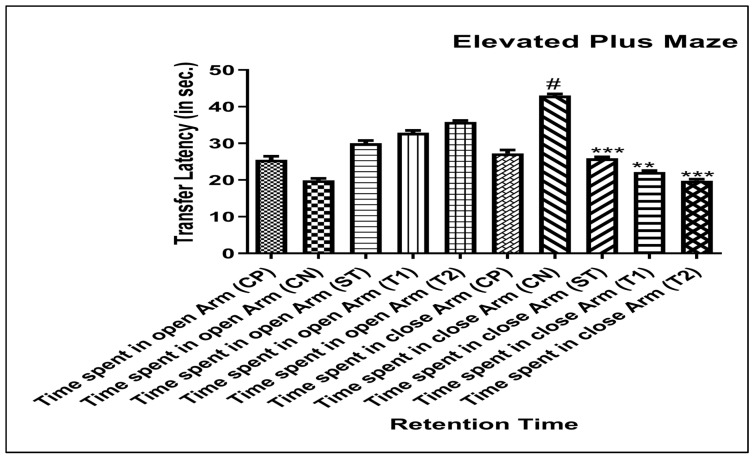
Effect of ALN-βCD nanoparticles using the Elevated Plus Maze model. Data are presented as mean ± SEM (*n* = 6), substantially different from the Control Negative (CN) group at *** *p* < 0.001. ** *p* < 0.1, # *p* < 0.01 vs. CP group. (Positive Control group (CP), Scopolamine Control group (CN), Standard Group (ST), Free Andrographolides (T1), ALN-βCD nanoparticles (T2)).

The memory of the experimental animals was significantly enhanced by the prepared formulation in comparison to the control group. When compared to the control group, the animals placed in the closed arm had a considerably shorter transfer latency time of 23.3 and 20.1 s, respectively, for time spent in free andrographolides (T1) and ALN-βCD nanoparticles (T2). This was in contrast to the open arm, which had a transfer latency time of 32.1 and 34.4 s, respectively (Figure 9). In the experimental analysis, the effect of EPM was evaluated based on the number of elevated entrances and the amount of time spent in open arms. An increase in both of these variables was indicative of the presence of an anxiety-reducing effect [19]. 

#### 2.10.2. Evaluation of Memory-Enhancing Potential by Using Y-Maze

**Figure 10:**
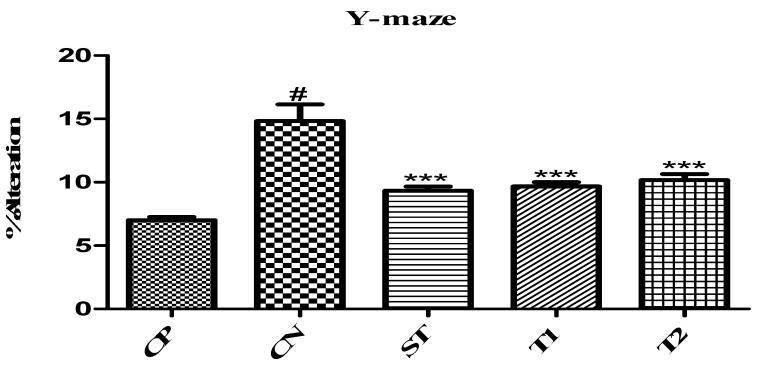
Effect of ALN-βCD nanoparticles using the Y-maze model. Data are presented as mean ± SEM (*n* = 6), substantially different from the Control Negative (CN) group at *** *p* < 0.001. # *p* < 0.01 vs. CP group. (Positive Control group (CP), Scopolamine Control group (CN), Standard Group (ST), Free Andrographolides (T1), ALN-βCD nanoparticles (T2)).

A rat’s propensity to explore unfamiliar areas can be measured using the Y-maze [20]. The experimental animals’ memory was much improved by the prepared formulation compared to the control group. Both the T1 and T2 test groups showed a very high percentage of change (9.43 and 10.23, respectively) (Figure 10). The experimental animals’ memory also improved significantly in the control group. These findings support earlier research showing that luteolin improves spatial recognition memory in the Y-maze test [21].

#### 2.10.3. Evaluation of Memory-Enhancing Potential by Using the Object Recognition Test

**Figure 11:**
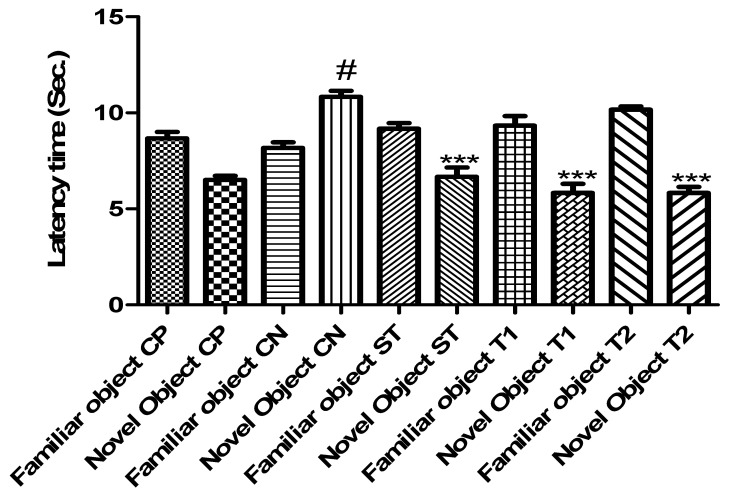
Effect of formulation on the latency time in the object recognition test. Data are presented as mean ± SEM (*n* = 6), substantially different from the Control Negative (CN) group at *** *p* < 0.001. # *p* < 0.01 vs. CP group. (Positive Control group (CP), Scopolamine Control group (CN), Standard Group (ST), Free Andrographolides (T1), ALN-βCD nanoparticles (T2)).

**Figure 12:**
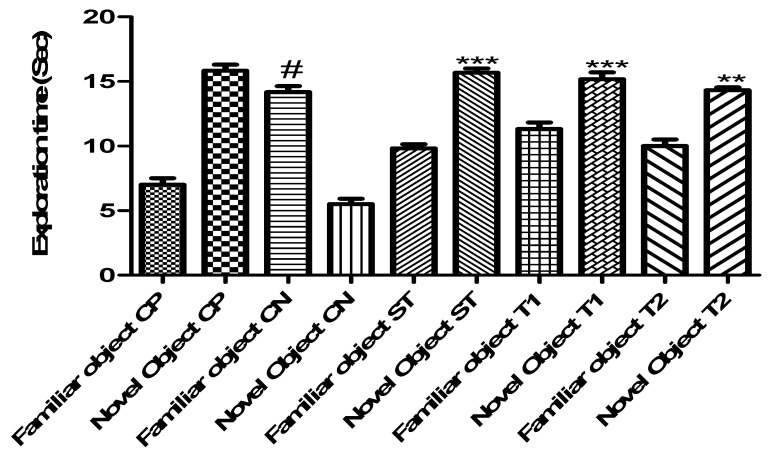
Effect of formulation on exploration time in the object recognition test. Data are presented as mean ± SEM (*n* = 6), substantially different from the Control Negative (CN) group at *** *p* < 0.001. ** *p* < 0.1. # *p* < 0.01 vs. CP group. (Positive Control group (CP), Scopolamine Control group (CN), Standard Group (ST), Free Andrographolides (T1), ALN-βCD nanoparticles (T2)).

**Figure 13:**
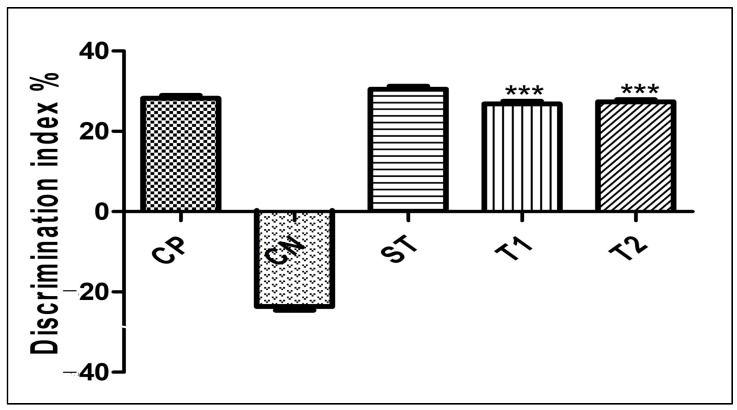
Effect of formulation on % discrimination index in the object recognition test. Data are presented as mean ± SEM (*n* = 6), substantially different from the Control Negative (CN) group at *** *p* < 0.001. vs. CP group. (Positive Control group (CP), Scopolamine Control group (CN), Standard Group (ST), Free Andrographolides (T1), ALN-βCD nanoparticles (T2)).

Figure 9 shows the efficacy of ALN-βCD nanoparticles on experimental models using the object recognition test. When compared to the control group, the experimental animals’ delay time was drastically reduced by the created formulation. In comparison to the control group, T1 took 5.43 s and T2 6.34 s (*p* < 0.001) (Figure 11). Yet, for T1 it was 14.1 s (*p* < 0.001) and for T2 it was 13.64 s, indicating a considerable increase in exploration time (Figure 12). While comparing T1 and T2 to the control group, the discrimination index for T1 was 27.4 s and 27.9 s, respectively (*p* < 0.001) (Figure 13). Neither the treated nor the untreated rats exhibited any deficits in locomotor activity, exploratory movement, or object recognition in the test [22].

#### 2.10.4. Evaluation of Motor Coordination Test by Using Rota-Rod

**Figure 14:**
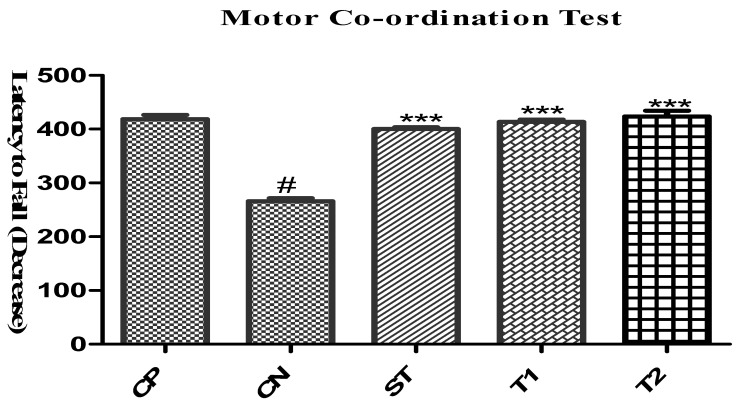
Effect of formulation on Motor coordination test. Data are expressed as mean ± SEM (*n* = 6), significantly different at *** *p* < 0.001 when compared with the Control Negative (CN) group. # indicates *p* < 0.01 when compared with the CP group. (Positive Control Group (CP), Scopolamine control group (CN), Standard Group (ST), Free Andrographolides (T1), ALN-βCD nanoparticles (T2)).

ALN-βCD nanoparticles showed significant motor coordination results. Compared to the control group, T1 had a delay to fall of 401 s and T2 of 412 s (Figure 14). When testing motor coordination with a rota-rod instrument, however, the control group demonstrated statistically significant results. Consistent with what Geng et al. found, we also found that andrographolide may protect neurons from parkinsonism disease, according to a 2019 report [23].

#### 2.10.5. Evaluation of Locomotor Activity by Using an Actophotometer

**Figure 15:**
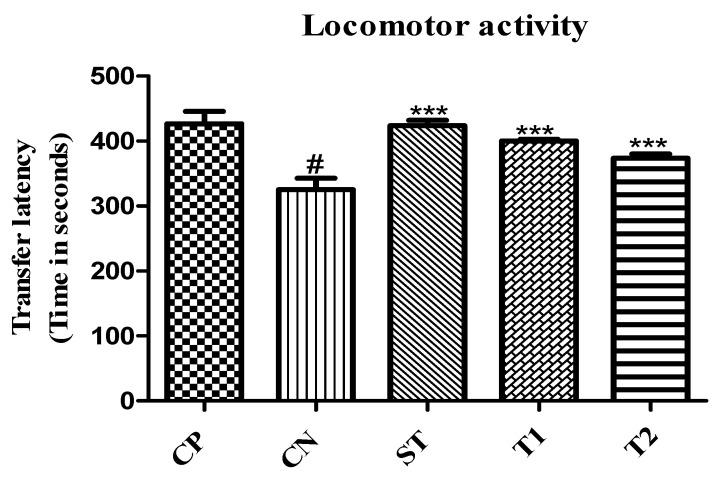
Effect of formulation on locomotor activity Data are presented as mean ± SEM (*n* = 6), substantially different from the Control Negative (CN) group at *** *p* < 0.001. # *p* < 0.01 vs. CP group. (Positive Control Group (CP), Scopolamine Control group (CN), Standard Group (ST), Free Andrographolides (T1), ALN-βCD nanoparticles (T2)).

Results showing the potency of nanoparticles for improving the locomotor activity of animals in the actophotometer model. The transfer latency was significantly reduced in treated groups. It was 390.44 and 378.43 s (*p* < 0.001) for T1 and T2, respectively (Figure 15).

### 2.11. Assessment of Biochemical Estimation

#### 2.11.1. Effect on Acetylcholinesterase Level

**Figure 16:**
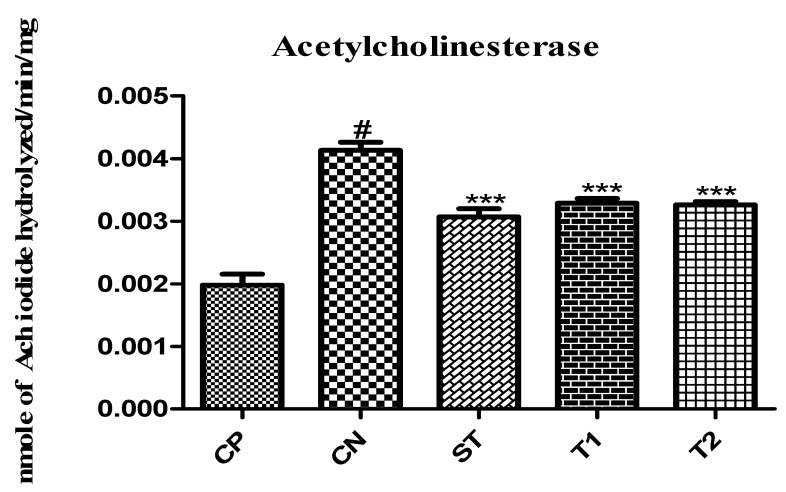
Effect of formulation on acetylcholinesterase level in animals. Data are presented as mean ± SEM (*n* = 6), substantially different from the Control Negative (CN) group at *** *p* < 0.001. # *p* < 0.01 vs. CP group. (Positive Control Group (CP), Scopolamine Control group (CN), Standard Group (ST), Free Andrographolides (T1), ALN-βCD nanoparticles (T2)).

The experimental animals’ acetylcholinesterase activity was substantially reduced by ALN-βCD nanoparticles (*p* < 0.001), leading to an increase in the quantity of acetylcholine. The enzymatic level of T1 and T2 was 0.0032 and 0.0030 Ach iodide hydrolysed/min/mg, respectively, in comparison to the control group (Figure 16). 

#### 2.11.2. Effect on Lipid Peroxidation

**Figure 17:**
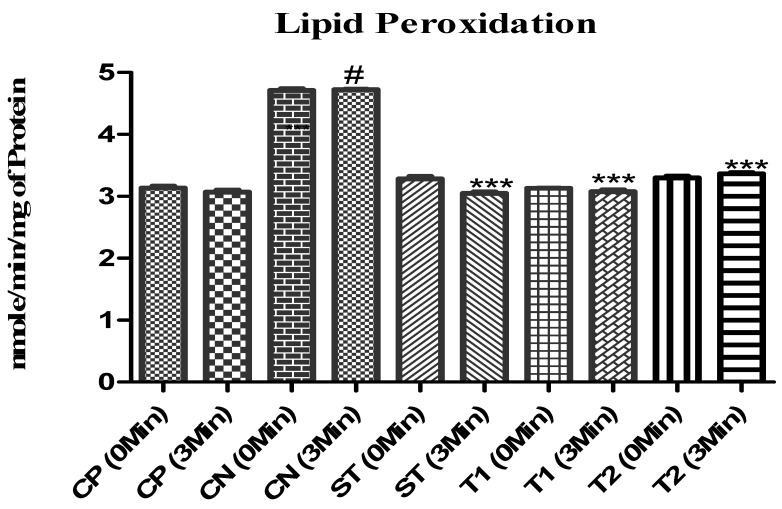
Effect of formulation on lipid peroxidation in animals. Data are presented as mean ± SEM (*n* = 6), substantially different from the Control Negative (CN) group at *** *p* < 0.001. # *p* < 0.01 vs. CP group. (Positive Control Group (CP), Scopolamine control group (CN), Standard Group (ST) group, Free Andrographolides (T1), ALN-βCD nanoparticles (T2)).

There was a significant decrease in lipid peroxidation activity (*p* < 0.001) following 3 min of observation. Comparing T1 and T2 to the control group, the enzymatic levels were 3.01 and 3.22 mmole/min/mg of protein, respectively (Figure 17).

### 2.12. Assessment of Histopathological Examination

**Figure 18:**
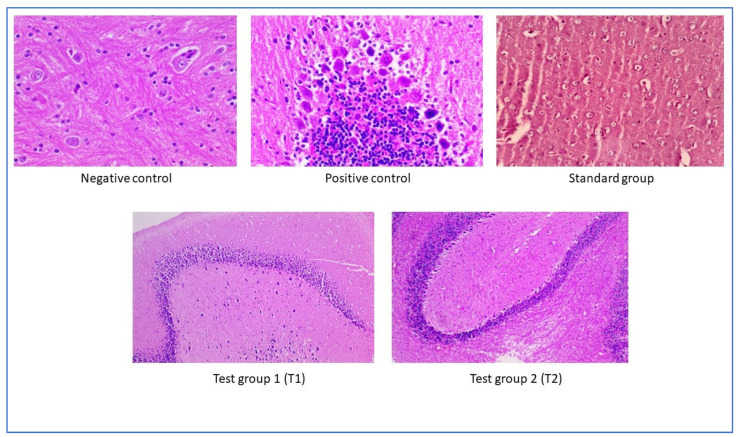
Histopathological analysis of mice brain (Scale bar of 50 μm).

The results exhibited that nanoparticle treatment in experimental animals improved memory cell functioning; no signs of toxicity were seen after treatment. Although the cells were more intact with the standard, a change in cell shape was seen after treating with free andrographolides. Based on the study results, we conclude that the prepared nanoparticles improved neuroprotective action (Figure 18).

## 3. Materials and Methods

### 3.1. Chemicals and Reagents

Andrographolides with a purity level of 98 ± 1 HPLC grade were sourced from Xi’an Lanyor Biotech Co., Ltd. of Shaanxi, China. The following ingredients were used: β-cyclodextrin, D-lactose monohydrate, and Polyvinyl alcohol (PVA) from Sigma-Aldrich. Dulbecco’s Modified Eagle Medium (DMEM) and 3-(4,5-dimethylthiazol-2-yl)-2,5-diphenyl tetrazolium bromide (MTT) was obtained from Genetix Biotech Asia Pvt. Ltd. of Mumbai, India, and phosphate buffered saline (PBS) from Sigma-Aldrich. NCCS in Pune, India, provided MDCKII endothelial cells and U373MG glial cells. There was no additional purification process before using any of the compounds. No additional purification was performed on any of the compounds before their direct usage.

### 3.2. Synthesis of Lactose-Appended β-Cyclodextrin

Lactose-appended β-cyclodextrin was synthesised by a previously reported method with minor modifications [24,25]. Equimolar amounts (7.5 g) of β-cyclodextrin and D-lactose monohydrate were taken in a mortar and pestle, and 3% activated carbon was added to the mixture and triturated. Furthermore, the triturated mixture was placed in a forced convection oven at 180 °C for 1 h. The heated sample was collected and dissolved in 30 mL of water. Lactose-appended β-cyclodextrin was obtained from filtration, and the practical yield was calculated to be 32%. The degree of substitution of lactose and β-cyclodextrin was analysed using HR-NMR.

### 3.3. Preparation of Andrographolide-Loaded Nanoparticles (ALN)

Nanoparticles of ALN-βCD were synthesised by the solvent evaporation technique. Lactose-appended β-cyclodextrin (50 mg) was dissolved in 5 mL of distilled water with continuous magnetic stirring (800 rpm) at 37 °C for 1.5 h. The organic phase was produced by adding 25 mg of andrographolides to 5 mL of chloroform. To prepare the aqueous phase, 0.01% (*w*/*v*) PVA was added to distilled water. The organic phase was combined with the water phase that contained PVA as a co-surfactant to make the emulsion more stable and to lower the surface tension. The mixture was subjected to sonication in a bath sonicator for 12 min. To remove the organic solvent, the produced emulsion was allowed to stand at room temperature for 24 h with magnetic stirring. A 25 min ultracentrifugation run at 4 °C and 20,000 rpm was used to collect the prepared nanoparticles. The nanoparticles were subsequently rinsed with distilled water and centrifuged once more. Before being stored at −20 °C until later usage, they were lyophilised and resuspended in distilled water [26].

### 3.4. Optimization of Formulation

The optimal nano formulation was achieved using the Box-Behnken Design (BBD) in the Design-Expert program. The design expert optimised the nano formulation over seventeen trial runs using a three-factor, three-level BBD. A study was conducted to determine the influence of three independent variables on the effect of particle size (nm), entrapment efficacy (%), and drug release (%). The variables included polymer concentration, surfactant concentration, and sonication time. Table 1 shows the effects of the three independent variables on R^1^, R^2^, and R^3^: high (+1), intermediate (0), and low (−1). To optimize the nano formulation utilizing Design-Expert (Version 12) software, the design explains the major use of quadratic effects of components A, B, and C on various selected responses [27].

### 3.5. Characterization of ALN-βCD Nanoparticles

The Anton Paar LitesizerTM 500 particle size analyser was used to check the nanoparticles’ zeta potential, polydispersity index (PDI), and size. The correct volume of sample was dissolved in deionised water, transferred to a cuvette, and then put in the particle size analyser’s sample holder. Analysis was performed to obtain the sample’s mean particle size and PDI once the necessary intensity was obtained. The zeta potential was determined using the electrophoretic light scattering method at 25 °C in triplicate (*n* = 3), with a separate sample container for particle size and PDI measurements. For the TEM imaging process, however, 0.2 mg of nanoparticles were diluted in 6 mL of distilled water. Afterwards, a micropipette was used to extract 2 µL of the diluted sample, which was then placed onto a carbon-coated copper grid. An infrared lamp was used to dry the samples for 5–6 min before they were inspected using a TEM [28].

### 3.6. Drug Loading and Entrapment Efficacy

A standard curve of AG was prepared using different concentrations (5–30 µg/mL) in methanol using a UV-visible spectrophotometer. Absorbance at 224 nm was measured for several AG solutions. The UV method was validated by plotting the graph between absorbance and concentration of AG. AG was isolated from ALN-βCD nanoparticles by preparing a solution (1 mg/1 mL) of ALN-βCD nanoparticles and methanol. The solution was placed in an orbital shaker for 20 h. The resulting mixture was centrifuged at 15,000 rpm for 30 min, and the supernatant was subjected to UV-visible spectrophotometry for measuring the absorbance at 224 nm [29,30]. The drug loading and entrapment efficacy % were estimated by the following equations:

$$\mathrm{Drug}\;\mathrm{loading}\;(\%)=\frac{\mathrm{Initial}\;\mathrm{Drug}-\mathrm{Free}\;\mathrm{Drug}}{\mathrm{weight}\;\mathrm{of}\;\mathrm{Nanoparticle}}\times 100$$

$$\mathrm{Drug}\;\mathrm{entrapment}\;(\%)=\frac{\mathrm{Total}\;\mathrm{Drug}-\mathrm{Free}\;\mathrm{Drug}}{\mathrm{Total}\;\mathrm{Drug}}\times 100$$

### 3.7. Fourier Transform Infrared Spectroscopy (FT-IR)

To evaluate the FT-IR spectra of free AG, lactose-appended β-cyclodextrin, and ALN-βCD nanoparticles, a Tensor 37 instrument from Bruker, Massachusetts, United States, was utilised. Samples weighing 5 mg were placed directly into the laser beam to obtain spectra in the scanning range of 4000–400 cm^−1^.

### 3.8. In Vitro Drug Release 

The dialysis membrane approach was used in two different pH ranges to determine the AG in vitro release profile. ALN-βCD nanoparticles were examined for AG release in vitro during dilution in PBS at physiological pH of 7.4 and acidic intracellular endosomal pH of 5.4. The dialysis bag included 10 mL of 0.2% (*w*/*v*) Tween 20, 2 mg ALN-βCD nanoparticles, and pH 7.4 and 5.4 PBS solutions. At 0, 30, 60, 120, 180, 360, 480, 720, 840, 1080, and 1440 min, 2 mL of the released media was collected from the dialysis bag and put into an equal volume of new PBS to keep the volume constant. The bag was placed in its own container. Using a UV-VIS spectrophotometer at a maximum wavelength of 224 nm, the concentration of AG was determined [31].

### 3.9. Cytotoxicity Assay 

A cytotoxicity study of ALN-βCD nanoparticles was performed by using U373MG cell viability assays. After a 24-h incubation at 37 °C in a humidified incubator with 5% CO_2_, 10,000 cells/well were seeded onto 96-well plates. Then, 100 µL of MTT solution was added to each well and incubated for three hours at 37 °C in a humidified incubator with 5% CO_2_. Following this, 100 µL of DMSO was added to every well, and the contents were carefully combined. To determine the MTT’s absorbance, a spectrophotometer was used at 550 nm [32].

### 3.10. In Vitro BBB Permeability

Transwell inserts were used in a co-culture model of two different cell lines (U373MG cells) to conduct an in vitro BBB permeability research. As a first step in creating the co-culture model, U-373 MG cells were seeded onto inserts covered with a 2% gelatin solution (Corning, New York, NY, USA) at a density of 7.5 × 10^4^ cells per well and given 30 min to proliferate. After that, the inserts were submerged in DMEM in 12-well plates for 24 h. A density of 150 × 10^4^ cells per well of U373MG cells were seeded onto the inner surface of the inserts and then incubated at 37 °C for 24 h. In subsequent steps, three different formulations, namely free AG, ALN-βCD nanoparticles, and β-Cyclodextrin, were diluted simultaneously and introduced to the luminal chamber of the inserts using serum-free DMEM. The concentration of these nanoparticles was 1 mL/well, meaning 2 mg/mL. At various time periods (0, 1, 12, 24, 48 h) after treatment with lactose-appended β-cyclodextrin nanoparticles, 200 µL of media was removed from the basal chamber. Equal volumes of serum-free DMEM were also added at each measurement to keep the sink condition constant. 

### 3.11. In Vitro Cellular Uptake

The in vitro cellular uptake experiment was performed using 90% confluent U373MG cells. The cells were exposed to ALN-βCD nanoparticles at a concentration of 100 μg/mL for four hours. In addition, after each wash, 2 mL of PBS at 4 °C was used. A cell lysis buffer was used to lyse the cells later on. Twenty minutes of centrifugation at 10,000 rpm was applied to the resultant lysate. Lastly, a plate reader (BMG Fluostar) equipped with 490/525 nm excitation and emission wavelengths was used to examine the lysate’s fluorescence.

### 3.12. Experimental Animals

The Animal House at Galgotias University in Greater Noida, Uttar Pradesh, India, provided Swiss Albino mice that ranged in weight from 20 to 25 g and had an age range of 8 weeks. Mice were kept in climate-controlled conditions in polypropylene cages covered with steel mesh. A relative humidity of 55 ± 5% and a temperature of 25 ± 5 degrees Celsius were maintained. Aside from having unlimited access to food and water, they were also subjected to regular 12-h light and dark cycles. A constant temperature of 25 ± 4 degrees Celsius was maintained in the room. The feeding was conducted in the Central Animal House. All of the animals were treated with kindness and compassion, following the guidelines set out by the Institutional Animal Ethics Committee (IAEC). The research has been approved by the Institutional Animal Ethics Committee in accordance with Regulation GU/IAEC/2022/04/03.

### 3.13. Treatment Protocol

The mice weighed between 25 and 30 g, and six rodents were randomly assigned to each of five groups (*n* = 6). Prior to dosing, they were maintained in their separate cages for 7 days to allow for acclimatisation to the study facility circumstances and to facilitate individual identification. Daily training trials lasting 15 min spaced 30 min apart were used to train the animals to accomplish tasks involving mazes. Training an animal to its full potential takes seven days, during which they do not receive any medicine. For the investigation, the researchers selected fully trained animals. In the first group, known as the control, mice were given distilled water. In the second group, known as the disease-induced group, mice took 1 mg/kg/day/i.p. of scopolamine for 21 days straight. In the third group, known as the standard group, mice took 1 mg/kg/day/i.p. of donepezil for 14 days straight, followed by 200 mg/kg/p.o. once a day for the next 14 days. In the test groups, T1 and T2, mice took 1 mg/kg/day/i.p. of free andrographolides and ALN-βCD nanoparticles for 14 days, respectively, followed by 100 mg/kg/p.o. once a day for the following 14 days. Following daily medication administration for one hour, all animals were evaluated using the Elevated Plus Maze and Y-Maze tests. A single investigator, unaware of the experimental settings, documented the successful trials and swimming trajectories in all behavioural models.

### 3.14. Behavioural Assessment of Prepared Formulation

#### 3.14.1. Elevated Plus Maze Test

The plus-maze was set up with two open arms set opposite each other, measuring 50 × 10 × 40 cm, and two enclosed arms measuring 50 × 10 × 40 cm, with an open canopy. The labyrinth was elevated to a height of 50 cm. Mice weighing 20–25 g were housed in pairs for 10 days before being tested in the device. To keep the mice from becoming overly stressed, the investigator would handle them every other day throughout this time. Six mice per dose were used in the experiments. Thirty minutes after receiving the test medication or standard orally, the mice were placed in the labyrinth facing one of the enclosed arms. The number of arm entries, time spent in each arm (open and enclosed), and number of entries into each arm were measured throughout a 5-min test. The procedure took place in a sound-attenuated room, and a TV camera controlled from a distance allowed observers to watch from another room [33,34].

#### 3.14.2. Y-Maze Test

Participants’ immediate spatial working memory was assessed using the Y-maze. The Y-maze had three equal-angle arms that were 30 cm long, 5 cm wide, and 12 cm high. Animals were placed in one arm on the sixth day after Nymphaea lotus seed extract treatment, and their arm entries were manually recorded for 8 min. The alternation score for each mouse was determined as a percentage by dividing the real number of alternations by the feasible number (all arm entries minus two) and multiplying by 100. The number of arm entries was considered when assessing locomotion [35,36].

#### 3.14.3. Object Recognition Test

The approach outlined by Ennaceur and Delacour [35] was used to conduct the object recognition test. An open field box measuring (50 × 50 × 40 cm) was used to carry out the three stages of this test. The habituation phase concluded with 5 min of free exploration of the open field by the mice. In the first phase of acquisition (T1), two red cubes measuring 444 cm were set up in opposite corners of the open area, 10 cm away from the sidewall. After positioning themselves in the centre of the wide field, the mice were given five minutes to investigate these two visually similar objects. Afterwards, they were returned to their cages. The test “choice” (T2) was made twenty-four hours following T1. The blue cone was introduced during T2, and the mice were exposed to both the familiar and the unfamiliar object [36]. In both T1 and T2, a stopwatch was used to manually record the amount of time the mice spent exploring each object. Afterwards, a discrimination index (DI) was computed using the following steps:

$$\mathrm{DI}=\frac{\mathrm{TN}-\mathrm{TF}}{\mathrm{TN}+\mathrm{TF}}\times 100$$

To measure the mice’s interest in novel items, we employed the discrimination index (DI). In this context, “time spent with the novel object” (TN) and “time spent with the familiar object” (TF) refer to the total amount of time spent on both types of objects; the DI is the proportion of time spent on the former relative to the total amount of time spent on both of them.

#### 3.14.4. Motor Coordination Test by Rota-Rod Apparatus

The purpose of the test is to determine the extent to which a drug is working to impair motor coordination. The capacity of an animal to stay on a spinning rod could be used to assess the degree to which a test substance relaxes skeletal muscles. We defined the endpoint as the dose at which half of the mice lost their capacity to stay on the rotating rod [36].

#### 3.14.5. Locomotor Activity Test by Actophotometer

An actophotometer was used to track the animal’s movement patterns. The basal activity score was recorded over a 5-min period after each animal was placed individually in the actophotometer. After 30 min and 1 h of treatment, each animal was given the corresponding medicine, and their activity score was recorded. An indicator of central nervous system depression was a decrease in activity score [35].

### 3.15. Biochemical Estimation

#### 3.15.1. Homogenization of Brain

Animals were rendered unconscious and then had their heads severed on the 22nd day using an intraperitoneal injection of ketamine (60 mg/kg) and xylazine (10 mg/kg). After that, the heads were cut open to remove the brains. The hippocampus was dissected on a dish that was placed on ice, and the tissues were stored for a number of different biochemical tests. After that, the tissues were rinsed in an ice-cold phosphate buffered saline solution that had a pH of 7.4 and included 10% by weight of phosphate buffer. The mixture was mixed and then spun at 4 °C (10,000 RPM) for 15 min to separate the components. The resulting liquid was then frozen at −20 °C [37,38].

#### 3.15.2. Protein Quantification

The biuret method was applied to provide an accurate reading of the protein content. To determine the value of the protein, a standard curve based on bovine serum albumin was utilised. The value of the protein was utilised in the process of normalizing the estimated values of several different biochemical parameters [39].

#### 3.15.3. Estimation of Acetylcholinesterase Activity 

A volumetric flask with a capacity of 25 mL was used to dilute 0.5 mL of the brain homogenate’s supernatant liquid with newly prepared dithiobis nitric benzoic acid (DTNB) solution. To fill two test tubes, two amounts of 4 mL were pipetted from the volumetric flask. The acetylcholine solution was introduced to one of the test tubes with two drops. After that, 1 mL of substrate solution was pipetted into each of the two test tubes and left to incubate at 30 °C for 10 min. Spectrophotometry, which measures changes in absorbance over time, was performed at 412 nm [40]. To determine the enzyme activity, the following formula was used:

R = [C × Volume of Assay (3 mL)/E × mg of protein]

where C = change in absorbance/minute.

R = rate of enzyme activity in ‘n’ mole of acetylcholine iodide hydrolysed/minute/mg protein

E = extinction coefficient (13,600/mL/cm).

#### 3.15.4. Determination of Lipid Peroxidation

A 1 mL suspension medium was extracted from the supernatant of the 10% tissue homogenate. After that, half a millilitre of 30% trichloroacetic acid (TCA) and half a millilitre of 0.8% thio barbituric acid (TBA) were transferred to it. After 30 min at 80 °C in a shaking water bath, the tubes were wrapped with aluminium foil. After 30 min, the tubes were removed and immersed in cold water for a further fifteen minutes. After that, they went through a 15 min centrifugation run at 3000 rpm. The supernatants’ absorbance was measured at 540 nm when the samples were at room temperature, in comparison to a suitable blank. One millilitre of distilled water, half a millilitre of a 0.8% TBA solution, and half a millilitre of a 30% TCA solution made up the blank [41]. A sample’s malondialdehyde (MDA) concentration was determined using the following formula:

$$\mathrm{MDA}\;\mathrm{level}\;(\mathrm{nmol})=\frac{\mathrm{Volume}\;\mathrm{of}\;\mathrm{test}\;\mathrm{solution}-\mathrm{optical}\;\mathrm{density}}{0.156}$$

### 3.16. Estimation of Protein

After centrifuging 10% tissue homogenate at 10,000 rpm, 5 mL of an alkaline solution was added to 1 mL of suspension obtained from the supernatant. The mixture was then allowed to stand for 10 min. The solution was mixed by adding half a millilitre of diluted Folin’s reagent and shaking the tube. Extinction was measured at 750 nm after 30 min using the correct blank.

#### Preparation of Calibration Standard Curve of Protein 

Various volumes were collected from a 5 mL solution of bovine serum albumin (2 mg/mL) and placed into 6 test tubes. To bring the volume of each tube to 1 mL, distilled water was added to all of them. We used the same method to determine the protein concentration in the sample and the six tubes described above. The relationship between optical density and protein concentration was graphed. The protein concentration in each millilitre of the sample suspension was determined using the calibration standard plot.

### 3.17. Histopathological Examination

Microscopic study of hippocampus sections of rat brains, stained with haematoxylin and eosin, was performed at 10× magnification [42,43]. A microtome was used to slice blocks of paraffin beeswax tissue after rinsing the prepared tissue with xylene and immersing it in paraffin for one day. After deparaffinizing the tissue sample, it was set on a glass plate and stained with eosin and hematoxylin so that it could be examined under a light microscope for histological purposes.

### 3.18. Statistical Analysis

All values were expressed as mean ± standard error of the mean (SEM). The statistical analysis was conducted using GraphPad Prism Version 8.0.1 and included a one-way analysis of variance (ANOVA) followed by Turkey’s *t*-test. As for the comparisons between the CN and CP groups, *** denotes *p* < 0.001 and # indicates *p* < 0.01, respectively.

## 4. Conclusions

This study evaluated the cognition enhancement efficacy of ALN-βCD nanoparticles on animals. By employing non-invasive delivery methods, these innovative nanoparticles exceed their comparable solution form, enabling a decrease in the dosage that needs to be supplied. We were able to show the complex impacts of ALN-βCD nanoparticles by using histopathological and biochemical evaluations. Additionally, we demonstrated through neurobehavioural tests that they significantly enhanced both short-term and long-term spatial memory. To summarise, our animal preclinical study showed that ALN-βCD nanoparticles could be a safe, effective, and non-invasive way to treat memory loss. As a result, we are able to explore headfirst into exploring the potential of clinical trials according to our positive outcomes.

## Figures and Tables

**Table 1:** 17 experimental runs generated by the Box−Behnken design.

STD	Run	Factor 1 Polymer Concentration (mg)	Factor 2 Surfactant Concentration (mg)	Factor 3 Sonication Time (min)	Response 1 Particle Size	Response 2 EE%	Response 3 Drug Release %
16	1	35	0.5	12	378	70	85
17	2	40	1	12	389	76	73
15	3	45	1	12	398	73	76
7	4	35	1	15	398	76	78
1	5	35	0.5	12	387	68	75
8	6	48	1	15	387	72	64
5	7	45	1	10	345	75	61
13	8	40	1	12	378	73	79
6	9	53	1	10	489	78	67
14	10	45	1	12	408	73	73
3	11	29	1.5	12	317	74	81
11	12	40	0.5	15	285	74	62
10	13	47	1.5	10	278	72	62
4	14	50	1.5	12	257	71	78
12	15	40	1.5	15	287	82	80
9	16	40	0.5	10	398	75	65
2L	17	53	0.5	12	495	79	71

**Table 2:** Drug loading and entrapment efficacy of the prepared formulation.

Tube	Concentration (µg/mL)	Absorbance	Drug Entrapment (mg)	Drug Loading	Drug Entrapment %	Drug Loading %
1	5	0.128	3.90	1.09	78.08	21.91
2	10	0.238	7.40	2.59	74.07	25.92
3	15	0.356	11.16	3.83	74.43	25.56
4	20	0.524	16.51	3.48	82.57	17.42
5	25	0.625	19.73	5.26	78.92	21.07
6	30	0.745	23.55	6.44	78.51	21.48

## Data Availability

The data are available upon request from the corresponding author.

## Supplementary Materials

The following supporting information can be downloaded at: https://www.mdpi.com/article/10.3390/ph17070966/s1, Figure S1: NMR spectra of lactose-appended β-cyclodextrin; Figure S2: Particle size, zeta potential, and TEM of the ALN-βCD nanoparticles; Figure S3: FTIR Spectrum, (A) Andrographolide-loaded Lactose β-Cyclodextrin nanoparticles (B) Lactose appended β-cyclodextrin.
