# Supplementary material for: Enhancement of Cognitive Function by Andrographolide-Loaded Lactose β-Cyclodextrin Nanoparticles: Synthesis, Optimization, and Behavioural Assessment
Source: Pharmaceuticals (Basel). 2024 Jul 21;17(7):966. doi: 10.3390/ph17070966 (PMC11279429; doi:10.3390/ph17070966)
Supplement: Supplementary file 1 [file pharmaceuticals-17-00966-s001.zip › pharmaceuticals-3101813-supplementary.pdf]

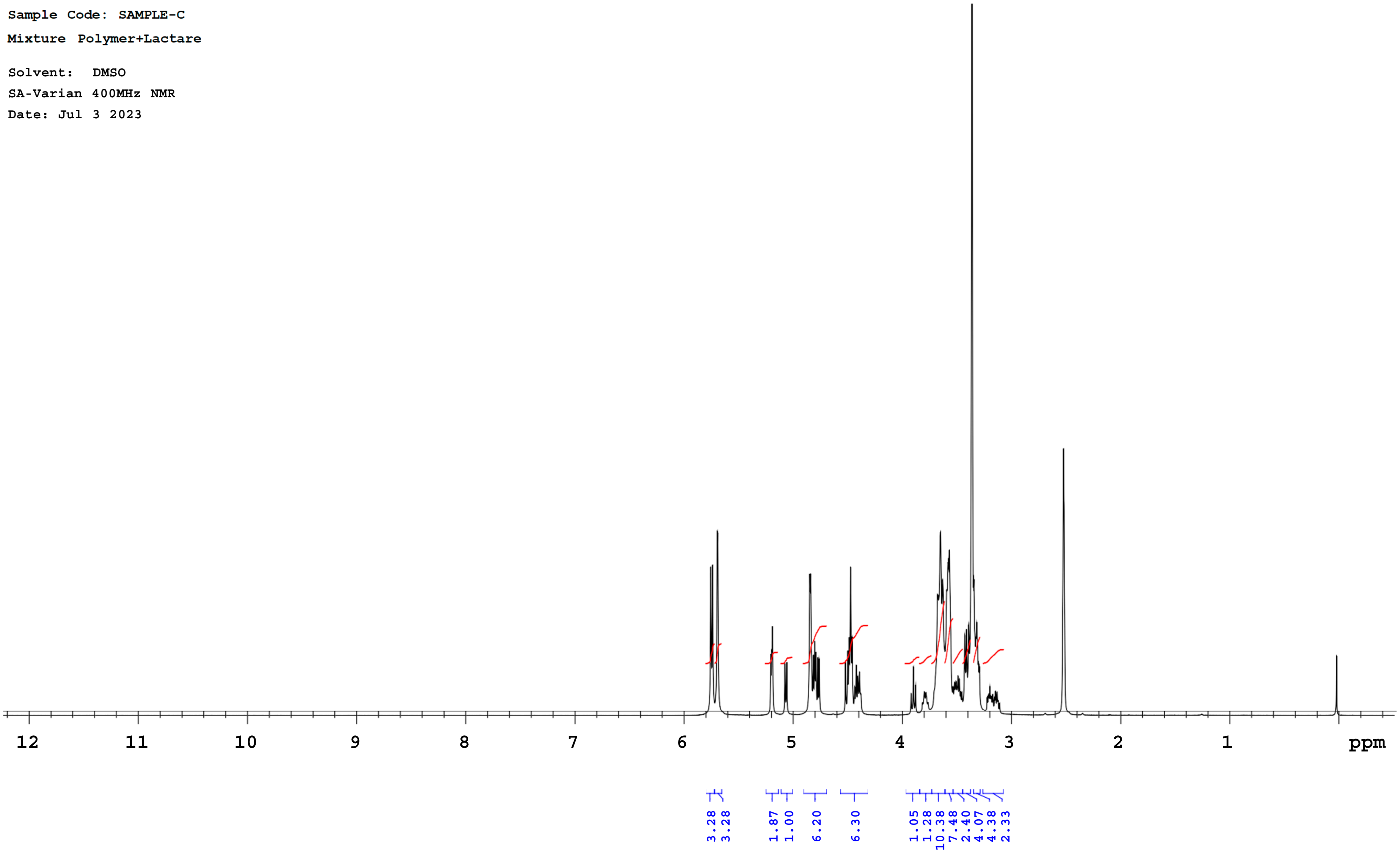


**Figure S1: NMR spectra of lactose added β-cyclodextrin**

Sample Code: SAMPLE-C
Mixture Polymer+Lactare

Solvent: dmso
SA-Varian 400MHz NMR
Date: Jul 3 2023

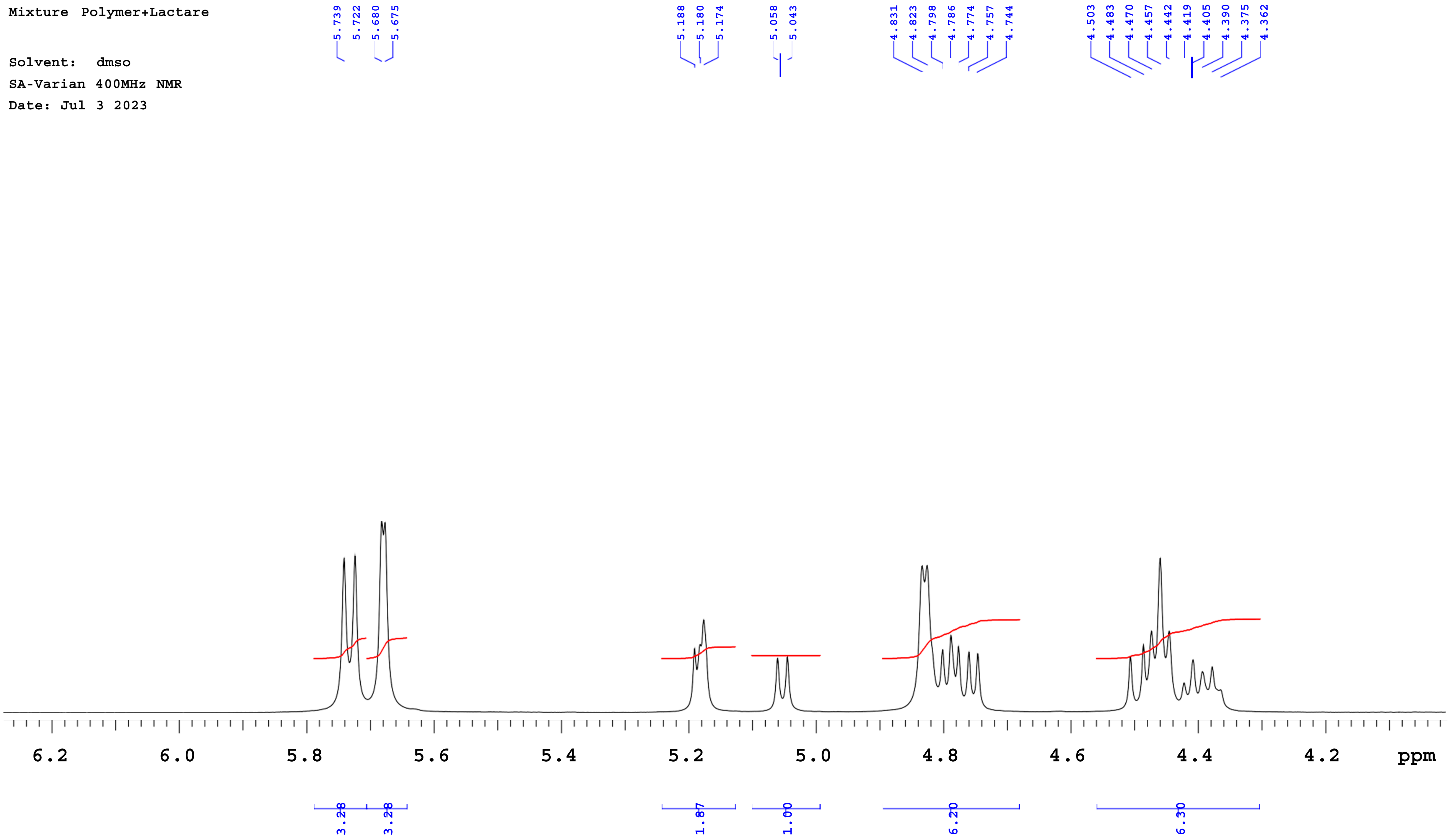


Plotname: SAMPLE-C_PROTON_20230703_01_plot02

Sample Code: SAMPLE-C

Mixture Polymer+Lactate

Solvent: dmso

SA-Varian 400MHz NMR

Date: Jul 3 2023

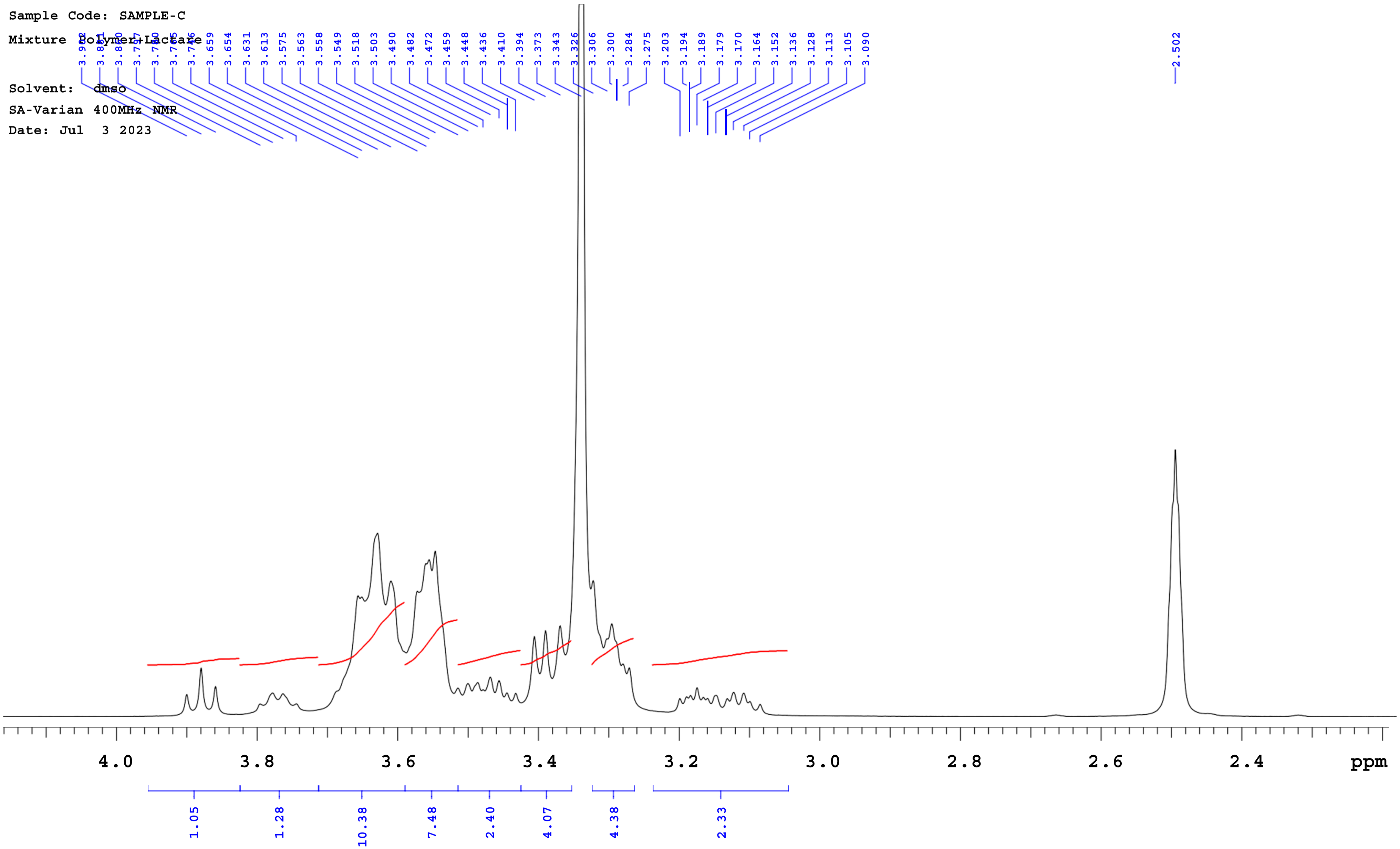


Plotname: SAMPLE-C_PROTON_20230703_01_plot03

Mixture Polymer+Lactare

exp2 PROTON

| SAMPLE | | PRESATURATION | |
|---|---|---|---|
| date | Jul 3 2023 | satmode | n |
| solvent | dmso | wet | n |
| file /home/varian/~ | | SPECIAL | |
| data/2023/Jul/SAMP~ | | temp | 25.0 |
| LE-C_20230703_01/S~ | | gain | 14 |
| AMPLE-C_PROTON_202~ | | spin | 20 |
| 30703_01.fid | | hst | 0.008 |
| ACQUISITION | | pw90 | 16.400 |
| sw | 7183.9 | alfa | 10.000 |
| at | 4.000 | FLAGS | |
| np | 57472 | il | n |
| fb | 4000 | in | n |
| bs | 2 | dp | y |
| d1 | 1.000 | hs | nn |
| nt | 128 | PROCESSING | |
| ct | 64 | lb | 0.50 |
| TRANSMITTER | | fn | not used |
| tn | H1 | DISPLAY | |
| sfrq | 399.691 | sp | -786.0 |
| tof | 799.4 | wp | 7183.7 |
| tpwr | 61 | rfl | 786.2 |
| pw | 8.200 | rfp | 0 |
| DECOUPLER | | rp | -119.2 |
| dn | C13 | lp | 0 |
| dof | 0 | PLOT | |
| dm | nnn | wc | 268 |
| decwave | W40_GATB-0~ | sc | 0 |
| | 12 | vs | 4999 |
| dpwr | 34 | th | 2 |
| dmf | 29412 | ai | ph |

| INDEX | FREQUENCY | PPM | HEIGHT | INDEX | FREQUENCY | PPM | HEIGHT | INDEX | FREQUENCY | PPM | HEIGHT |
|---|---|---|---|---|---|---|---|---|---|---|---|
| 1 | 2293.9 | 5.739 | 28.6 | 33 | 1497.4 | 3.746 | 2.5 | 65 | 1259.9 | 3.152 | 4.1 |
| 2 | 2287.1 | 5.722 | 29.0 | 34 | 1462.5 | 3.659 | 23.3 | 66 | 1253.6 | 3.136 | 3.5 |
| 3 | 2270.2 | 5.680 | 35.5 | 35 | 1460.3 | 3.654 | 23.1 | 67 | 1250.1 | 3.128 | 4.7 |
| 4 | 2268.2 | 5.675 | 35.2 | 36 | 1451.3 | 3.631 | 35.3 | 68 | 1244.2 | 3.113 | 4.6 |
| 5 | 2073.7 | 5.188 | 11.8 | 37 | 1443.9 | 3.613 | 26.1 | 69 | 1240.9 | 3.105 | 3.0 |
| 6 | 2070.2 | 5.180 | 12.6 | 38 | 1429.0 | 3.575 | 24.1 | 70 | 1235.0 | 3.090 | 2.4 |
| 7 | 2068.0 | 5.174 | 17.2 | 39 | 1423.9 | 3.563 | 29.4 | 71 | 1000.2 | 2.502 | 51.3 |
| 8 | 2021.6 | 5.058 | 10.1 | 40 | 1422.2 | 3.558 | 30.2 | 72 | -0.0 | -0.000 | 11.5 |
| 9 | 2015.4 | 5.043 | 10.3 | 41 | 1418.7 | 3.549 | 31.8 | | | | |
| 10 | 1930.8 | 4.831 | 27.2 | 42 | 1406.0 | 3.518 | 5.6 | | | | |
| 11 | 1927.7 | 4.823 | 27.3 | 43 | 1400.3 | 3.503 | 6.4 | | | | |
| 12 | 1917.9 | 4.798 | 11.6 | 44 | 1394.8 | 3.490 | 6.6 | | | | |
| 13 | 1912.8 | 4.786 | 14.3 | 45 | 1391.7 | 3.482 | 5.2 | | | | |
| 14 | 1908.0 | 4.774 | 12.2 | 46 | 1387.5 | 3.472 | 7.7 | | | | |
| 15 | 1901.4 | 4.757 | 11.2 | 47 | 1382.7 | 3.459 | 7.0 | | | | |
| 16 | 1895.9 | 4.744 | 10.9 | 48 | 1378.1 | 3.448 | 4.6 | | | | |
| 17 | 1799.9 | 4.503 | 10.4 | 49 | 1373.3 | 3.436 | 4.6 | | | | |
| 18 | 1791.8 | 4.483 | 12.5 | 50 | 1362.8 | 3.410 | 15.4 | | | | |
| 19 | 1786.8 | 4.470 | 15.1 | 51 | 1356.4 | 3.394 | 16.6 | | | | |
| 20 | 1781.3 | 4.457 | 28.6 | 52 | 1348.1 | 3.373 | 17.5 | | | | |
| 21 | 1775.6 | 4.442 | 15.1 | 53 | 1336.2 | 3.343 | 362.2 | | | | |
| 22 | 1766.4 | 4.419 | 5.5 | 54 | 1329.4 | 3.326 | 26.2 | | | | |
| 23 | 1760.7 | 4.405 | 9.8 | 55 | 1321.6 | 3.306 | 15.0 | | | | |
| 24 | 1754.8 | 4.390 | 7.6 | 56 | 1318.9 | 3.300 | 17.9 | | | | |
| 25 | 1748.6 | 4.375 | 8.5 | 57 | 1312.6 | 3.284 | 10.2 | | | | |
| 26 | 1743.4 | 4.362 | 4.2 | 58 | 1309.1 | 3.275 | 9.4 | | | | |
| 27 | 1559.4 | 3.902 | 4.3 | 59 | 1280.3 | 3.203 | 3.5 | | | | |
| 28 | 1551.1 | 3.881 | 9.4 | 60 | 1276.6 | 3.194 | 3.9 | | | | |
| 29 | 1543.0 | 3.860 | 5.9 | 61 | 1274.4 | 3.189 | 4.1 | | | | |
| 30 | 1517.8 | 3.797 | 2.5 | 62 | 1270.7 | 3.179 | 5.6 | | | | |
| 31 | 1510.8 | 3.780 | 4.6 | 63 | 1267.2 | 3.170 | 3.8 | | | | |
| 32 | 1504.8 | 3.765 | 4.5 | 64 | 1264.8 | 3.164 | 3.6 | | | | |

Plotname: SAMPLE-C_PROTON_20230703_01_plot04

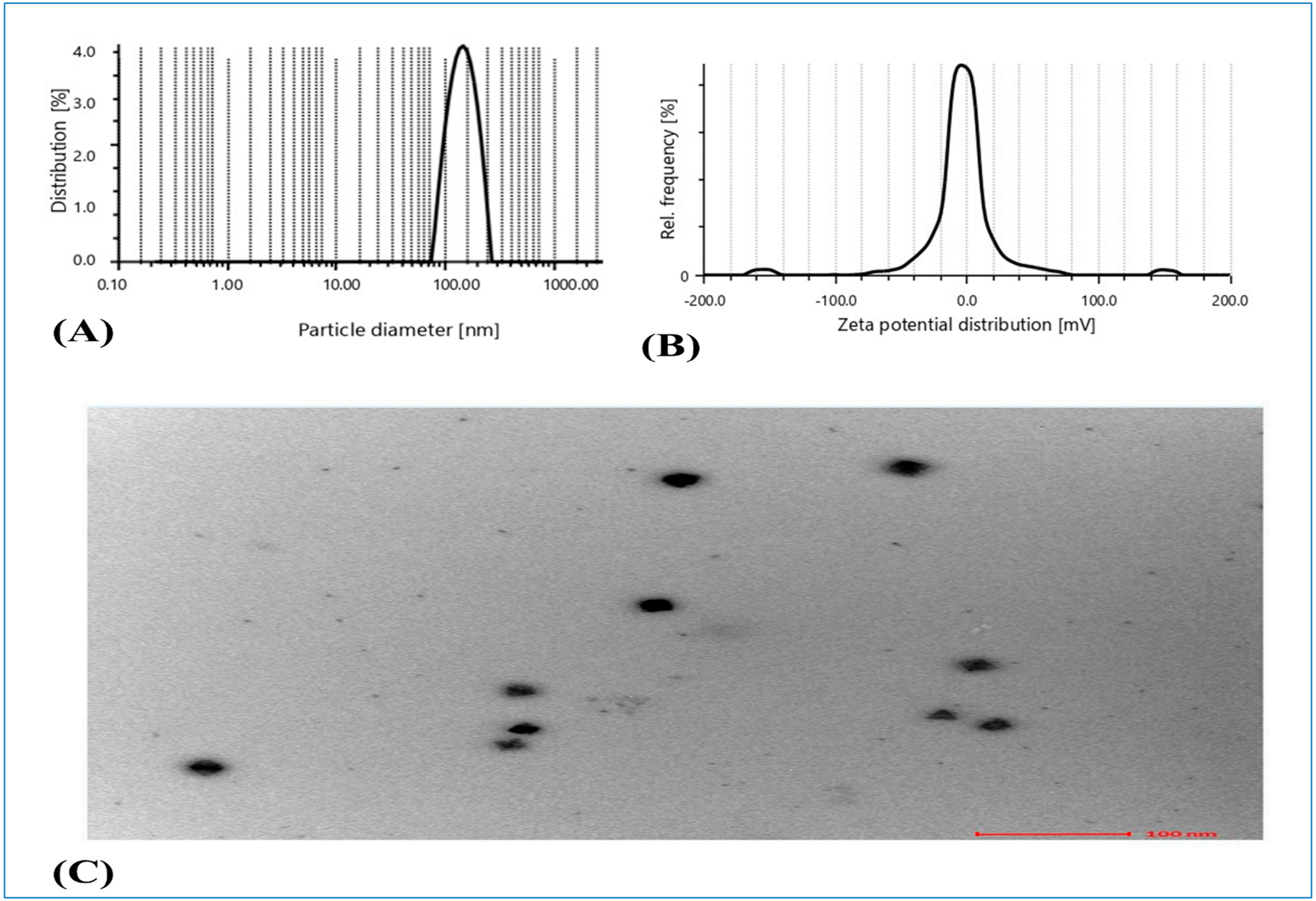


**Figure S2: Particle size (A), Zeta potential (B), and TEM of the ALN-βCD nanoparticles (C).**

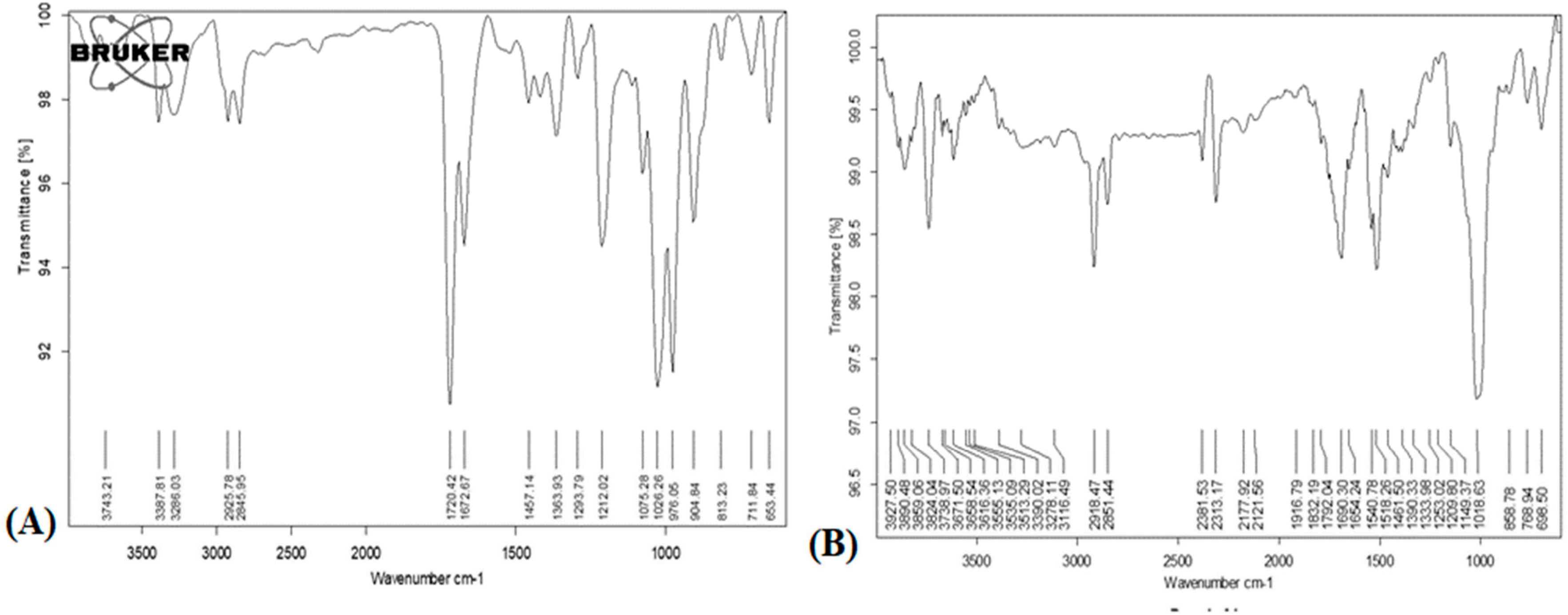


**Figure S3: FTIR Spectrum, (A) Andrographolide-loaded Lactose β-Cyclodextrin nanoparticles (B) Lactose appended β-cyclodextrin**
